# The effect of Ebola virus disease on maternal health service utilisation and perinatal outcomes in West Africa: a systematic review

**DOI:** 10.1186/s12978-022-01343-8

**Published:** 2022-02-04

**Authors:** Zemenu Yohannes Kassa, Vanessa Scarf, Deborah Fox

**Affiliations:** 1 Department of Midwifery, College of Medicine and Health Sciences, Hawassa University, Hawassa, Ethiopia; 2 Centre for Midwifery, Child and Family Health, Faculty of Health, University of Technology Sydney, Sydney, Australia

**Keywords:** Ebola virus disease, Maternal health, West Africa

## Abstract

**Background:**

Ebola outbreaks pose a major threat to global public health, especially in Sub-Saharan Africa. These outbreaks disrupt the already fragile maternal health services in West Africa. The aims of this study is to assess the effect of Ebola virus disease (EVD) on maternal health service utilisation and perinatal outcomes.

**Methods:**

This systematic review was conducted in West Africa, and the databases used were Medline, PubMed, CINAHL, Scopus, EMBASE and African journals online. Studies that reported the effect of the Ebola outbreak on maternal health services in West Africa were eligible for this systematic review. The search was limited to articles written in the English language only and published between 2013 and 2020. Three authors independently appraised the articles, and the data were extracted using a standardised data extraction format. The findings were synthesised using a narrative summary, tables, and figures.

**Results:**

Twelve studies met the inclusion criteria and were used for this systematic review synthesis. The results showed that antenatal care significantly decreased during Ebola virus disease and strove to recover post-Ebola virus disease. Women were less likely to have institutional childbirth during Ebola virus disease and struggled to recover post-Ebola virus disease. In addition, this review revealed a substantially higher rate of maternal mortality post EVD than those observed before or during the outbreak.

**Conclusion:**

Based on our findings, antenatal care, institutional childbirth, and postnatal care are attempting to recover post-Ebola virus disease. We recommended that responsible bodies and stakeholders need to prepare locally tailored interventions to increase the number of women attending ANC, institutional childbirth, and PNC services post-EVD and future outbreaks including COVID-19. In order to build trust, creating community networks between health care providers and trusted community leaders may increase the number of women attending antenatal care (ANC), institutional childbirth and postnatal care (PNC) post-EVD and during future outbreaks. Further studies are needed to examine health centre and hospital availability and accessibility, and capacity to deliver maternal health services post-Ebola virus disease and future outbreaks.

## Plain Language Summary

Ebola virus disease (EVD) is a serious public health concern affecting the health of humans and other primates. These outbreaks disrupt the already fragile maternal health services in West Africa. There is limited data on the effect of EVD on maternal health service utilisation and perinatal outcomes in West Africa. This systematic review aims to synthesise evidence on maternal health service utilisation and perinatal outcomes before EVD, during EVD and post EVD.

This systematic review was conducted in West Africa, and the databases used were Medline, PubMed, CINAHL, Scopus, EMBASE and African journals online. Twelve studies met the inclusion criteria and were used for this systematic review synthesis. The results showed that antenatal care significantly decreased during the Ebola virus outbreak and strove to recover post-Ebola virus disease. This finding indicated that women were less likely to have an institutional birth during EVD and struggled to recover post-Ebola virus disease. Based on this finding, responsible bodies and stakeholders need to prepare locally tailored interventions to increase the number of women attending ANC, institutional childbirth, and PNC services post-EVD and future outbreaks.

## Background

Ebola virus disease (EVD) is a serious public health concern affecting the health of humans and other primates [1]. The causative agent of Ebola is an RNA virus of the family Filoviridae, genus *Ebolavirus.* There are five known strains of the Ebola virus: Zaire Ebola virus (EBOV), Sudan Ebola virus (SUDV), Bundibugyo Ebola virus (BDBV), Forest Ebola virus (TAFV), and Reston Ebola virus (RESTV) [2–4]. Three of the above viruses are seriously pathogenic and lethal to humans. In contrast, the Reston virus is only pathogenic to non-human primates [5], and the natural reservoir of Ebola virus disease is in fruit bats [2, 6].

EVD is a virulent and extremely contagious viral haemorrhagic fever (VHF) [7], and its mode of transmission from person to person is via direct contact of the skin or mucous membranes with infected bodily fluids [1, 8, 9]. It was first discovered in 1976 in Zaire [10]. The 2014 Ebola outbreak posed a major threat to global public health, especially in West Africa. The first Zaire Ebola virus case was reported in December 2013 in Guinea, and it subsequently spread to Sierra Leone and Liberia [11]. The World Health Organization (WHO) declared the Ebola outbreak a public health emergency on August 8, 2014 [12]. From 2013 to 2016, 28,616 people had contracted EVD, and 11,310 people had died due to the Ebola virus disease in West Africa [13].

Similarly, maternal and neonatal deaths increased; directly by contracting the virus and indirectly through the overwhelming need for maternal health services [14]. In the last three decades, evidence showed that maternal mortality significantly decreased in three West African countries (Guinea, Liberia, and Sierra Leone) prior to EVD [15]. However, the EVD outbreak has reversed this tremendous progress in reducing maternal mortality [16–18]. Maternal and child health experts, policymakers and governments have implemented different intervention strategies to increase maternal health service utilisation in three West African countries before EVD (Guinea, Liberia, and Sierra Leone) [19]. These strategies include, for example, preparing maternal waiting rooms [20, 21], providing free health services [22], training and deploying midwives at health institutions [23], and community engagement in health [24].

Despite the above, interventions, maternal and neonatal morbidity and mortality are still high in these countries. In 2013, there were an estimated 650 maternal deaths per 100,000 live births recorded in Guinea, 640 maternal deaths per 100,000 live births recorded in Liberia, and 1100 maternal deaths per 100,000 live births recorded in Sierra Leone [25]. Furthermore, an estimated 30 perinatal deaths per 1000 live births occurred in Liberia in 2013 Demographic and Health Survey (DHS), and 39 perinatal deaths per 1000 live births occurred in Sierra Leone in 2013 DHS. The pooled estimated was 36 perinatal deaths per 1000 live births in West Africa [26].

Ebola virus disease has decreased institution-based childbirth [27, 28], devastating impact on the health system and health care providers [29, 30] and caused thousands of maternal and neonatal deaths. Due to these factors, there has also been an increase in maternal and neonatal morbidity and mortality through direct and indirect impacts on institutional childbirth [28, 31]. In 2014, the United Nations Population Fund projected that 120,000 maternal deaths could have occurred due to disruption of the Ebola outbreak if the necessary lifesaving emergency obstetrics care had not been urgently deployed across Guinea, Sierra Leone, and Liberia [30].

The EVD outbreak disturbed the already weak maternal health services [32, 33] due to ignorance, lack of supplies, or the shifting of health staff, equipment from the maternal service to EVD management [34, 35] and shut down public health facilities [36]. Additionally, medical health care providers' deaths, the absence of health care providers, the fear of being exposed to body fluids at health facilities [37, 38], and women’s belief that health facilities could be a source of Ebola transmission [39, 40], along with a negative attitude about the staff [41], has disrupted maternal health service utilisation.

While some systematic reviews have focused on determining the impact of the Ebola virus disease outbreak on maternal health service utilisation [28], these have not shown the effect of the Ebola virus disease outbreak on perinatal outcomes. A previous systematic review focused on barriers to maternal health services during the Ebola virus disease outbreak [42]. Therefore, this systematic review aims to synthesise evidence of the effect of the Ebola virus disease outbreak on maternal health service utilisation and perinatal outcomes.

## Methods

### Bibliographic data bases search strategies

**Fig. 1:**
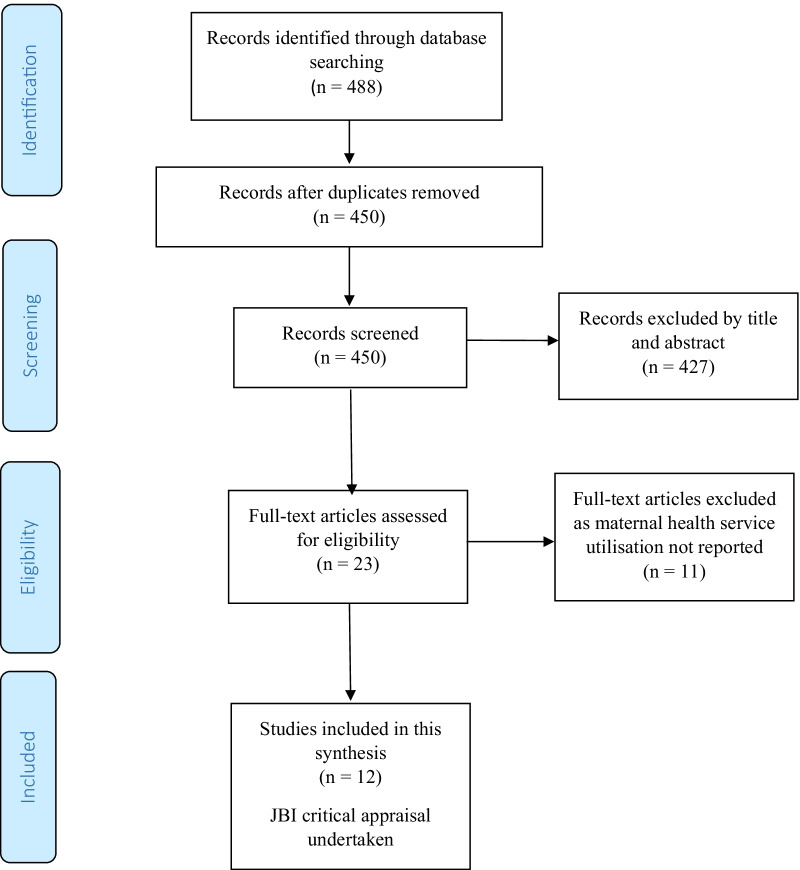
PRISMA (Flow chart of study selection for a systematic review of the effect of Ebola virus disease on maternal and neonatal health services utilisation in West Africa)

This systematic review was limited to peer-reviewed, published studies. The search strategy included the following databases: Medline, PubMed, CINAHL, Scopus, EMBASE and African journals online. Using special index search terms (medical subject headings (MeSH)) "Maternal health service" OR "reproductive health service" OR "maternal and new-born health service" OR "antenatal care" OR "postnatal care" OR "maternal primary care" OR "obstetrics care" OR "maternal-child health services" AND "Ebola*" OR "haemorrhagic fever" AND "utilisation*" OR "access" OR "uptake" OR "availability" AND "West Africa". In addition, additional articles were retrieved by using cross-referencing of references, titles, and abstracts. We registered our protocol with Prospero international register of systematic reviews (http://www.crd.york.ac.uk/PROSPERO/) in September 2020 (CRD42020202548). The Preferred Reporting Items for Systematic Reviews and Meta-Analysis (PRISMA) checklist [43] was utilised to present the findings on the impact of Ebola on obstetric care in West Africa (Fig. 1).

### Inclusion and exclusion criteria

Studies that reported the impact of the Ebola outbreak on maternal health services in West Africa were eligible for this systematic review. The search was limited to English language articles only, and articles published between 2013 and 2020 were included. Quantitative studies of cross-sectional, ecological, retrospective cohort and prospective cohort study designs in West Africa were included, irrespective of whether the study was implemented in a health facility and/or in the community.

Review articles, notes, editorial letters, commentaries, studies where the participants were not human, case reports, conference abstracts and proceedings, articles with incomplete information, articles with methodological problems or with full text not available and studies that reported the impact of Ebola on the health system without reporting its impact on maternal health services were excluded. When multiple publications of the same data exist, we used the most inclusive, comprehensive, and recent articles.

### Data quality appraisal

Three authors (ZYK, VS and DF) independently extracted data using a standardised data extraction format. The data extraction was performed using the Joanna Brigg's Institute (JBI) critical appraisal checklist for simple prevalence, which contains nine checklist items. The tool contains nine criteria to assess the quality of the studies, such as the appropriateness of the sampling frame and sampling method; adequacy of the sample size; complete descriptions of the study setting and participants, data analysis, statistical analysis, and response rate; the validity of the methods used to identify the condition; and the reliability of measurements between study participants [44]. Based on the above criteria, three authors (ZYK, VS and DF) independently assessed the quality of the articles. Any disagreement was resolved through discussion and consensus among the three authors. The quality of the study was evaluated, and studies that scored ≥ 5 out of 9 were included in this systematic review. Finally, the selected articles that met the inclusion criteria were retained for the narrative synthesis.

### Data synthesis

We employed a narrative synthesis approach to present the findings of this systematic review [45]. We evaluated the impact of EVD on different maternal health services, including antenatal care [1–4], facility-based childbirth, caesarean section, and postnatal care, which are all part of the continuum of care [46]. Finally, summary tables were produced from the crude data demonstrating the impact of the Ebola outbreak on maternal health services (Table 1).

**Table 1:** Evidence summary of a systematic review of the effect of the Ebola outbreak on maternal health services [16–18, 35, 39, 47–53]

Author, year Country	Study design	Study period	Study objective	Results	SQ
Brolin et al. 2016 [35] Sierra Leone	Cross-sectional study	From January 2014 to May 2015	To assess the potential impact of EVD on nationwide access to obstetric care	1. Pre-Ebola virus disease (EVD) outbreak hospital childbirth was 394, and hospital childbirth decreased by 312 (− 21%) during the outbreak 2. During EVD slow down hospital childbirth decreased by 283 (− 28%) 3. Pre-EVD caesarean section birth was 112, and caesarean section birth decreased by 89(− 20%) during peak the outbreak 4. During EVD slowdown caesarean section birth decreased by 89(-20%)	8/9
Camara et al. 2017 [47] Guinea	Ecological study	Pre-Ebola (1 March 2013 to 28 February 2014), during Ebola (1 March 2014 to 28 February 2015) and post-Ebola (1 March to 31 July 2016)	To compare trends in family planning, antenatal care, and institutional deliveries over the period before, during and after the outbreak	1. Pre-EVD average monthly ANC1 contact was 2053, and during EVD ANC1 contact declined by 59% (842) and similar trends ANC3 contact and above as ANC1 contact 2. During post-Ebola average monthly ANC1 contact increased by 1260 and recovered by 63% (recovery gap was 37%, p < 0.001 before Ebola) 3. Pre EVD average monthly institutional childbirth was 1223, during EVD institutional childbirth decreased by 62% (464) in October 2014 (during the peak EVD outbreak period) 4. During post-EVD average monthly institutional childbirth was 792 (recovery gap was 33%, p < 0.001) 5. Caesarean section significantly decreased during the peak of the EVD outbreak 6. A fully recovery was observed in post EVD caesarean section childbirth monthly mean caesarean section was (37, SD = 8) compared to the pre-Ebola level (38, SD = 7, p = 0.692) 7. Maternal deaths were low and remained similar across the three periods (0.1–0.2%, p > 0.05). Adverse new-born outcomes (deaths and stillbirths) were also low across the periods (range 1.1–1.7%) but were higher in the post-Ebola period compared to the pre-Ebola period (p < 0.01)	9/9
Caulker et al. [52] 2017 Sierra Leone	Cross-sectional study	From 2013 to 2015	To compare maternal health service utilization trends before, during, and after the Ebola outbreak (2014–2016)	1. Pre-EVD outbreak monthly ANC1 contact was (N = 1350 ± 109), and ANC1 contact did not significantly decrease during EVD (N = 1329 ± 159, p = 0.7), and ANC1 contact did not increase during post EVD (*N* = 1388 ± 127, p = 0.9) 2. Pre-EVD outbreak monthly ANC4 contact was (N = 1172 ± 52), and ANC4 significantly decreased during EVD outbreak (N = 1115 ± 76, p = 0.05), and ANC4 significantly increased during post EVD (N = 1131 ± 46, p = 0.05) 3. Pre-EVD outbreak monthly institutional childbirth was (N = 1109 ± 65), and institutional childbirth did not significantly decrease during EVD outbreak (N = 1090 ± 56, p = 0.5), and institutional childbirth did not significantly increase during post-EVD (N = 1127 ± 72, *p* = 0.2) 4. Pre-EVD outbreak monthly maternal postnatal care was (n = 1110 ± 51), and maternal postnatal care did not significantly decrease during EVD outbreak (N = 1105 ± 61,* p* = 0.8) and maternal postnatal care did not significantly increase post-EVD (n = 1165 ± 87,* p* = 0.09) 5. Pre-EVD outbreak monthly neonatal postnatal care was (N = 1028 ± 41), and neonatal postnatal care did not significantly decrease during EVD outbreak (N = 1050 ± 67,* p* = 0.4) and neonatal postnatal care did not significantly increase post-EVD (N = 1085 ± 114,* p* = 0.3)	8/9
Delamou et al. 2017 [16] Guinea	Retrospective cohort study	Pre-Ebola virus disease epidemic (January 2013 to February 2014), during the epidemic (March 2014 to February 2015) and post epidemic (March 2015, to February 2016)	To examine monthly service use data for eight maternal and child health service indicators	1. Pre-EVD ANC1 contact significantly increased by 9568 (9568, 95% CI 8941 to 10 195, p < 0.0001), and ANC3 and above contact significantly increased 7555 (7555, 95% CI 7098 to 8012, p < 0.0001) 2. Pre-EVD ANC1 contact increased per month by 109 (109, 95% CI 54 to 164, p < 0.0005), and ANC3 and above contact increased per month by 119 (95% CI 79 to 158, p < 0.0001) 3.During EVD ANC1 contact average monthly decreased by 923 (− 923, 95% CI − 1882 to 36, p = 0.0585), and ANC3 contact decreased per month by 624 (− 624, 95% CI − 1568 to 320, p = 0.1834) 4. Trends changed during versus pre-EVD ANC1 contact significantly decreased by 418 (− 418, 95% CI − 535 to − 300, p < 0.0001), and ANC3 contact and above contacts significantly decreased by 363 (− 363, 95% CI − 485 to − 242, p < 0.0001) 5. Pre-EVD institutional childbirth significantly increased by 3602 (3602, 95% CI 3345 to 3859, p < 0.0001) 6. Pre-EVD institutional childbirth increased per month by 61 (61, 95% CI 38 to 84, p = < 0.0001) 7. During EVD institutional childbirth decreased per month by 72 (72, 95% CI − 333 to 476, p = 0.7163) 8. During EVD institutional childbirth significantly decreased by 240 (− 240, 95% CI − 293 to − 187, p < 0·0001) 9. Post-EVD average monthly ANC1 attendance increased by 1712 (1712, 95% CI357 to 3066, p = 0·0157), and ANC3 contacts and above increased per month by 103 (103, 95% CI − 1385 to 1590, p = 0·8871) 10. Post-EVD institutional childbirth increased per month by 982 (982, 95% CI 362 to 1602, p = 0·0034) 11. Overall trend of post EVD ANC1 contact significantly declined by 136 (− 136, 95% CI − 231 to − 40, p = 0·0075), and ANC3 contact and above did not significantly decline by 13 (13, 95% CI − 109 to 134, p = 0.8286) 12.Overall trend of institutional childbirth did not significantly decline by 30 (− 30, 95% CI − 80, to 20, p = 0·2294)	7/9
Jones et al 2016 [17] Sierra Leone	Cross-sectional study	April 2013–January 2015	To determine the impact of the Ebola virus epidemic on routine maternity services	1. ANC1 and above contact significantly decreased by 18% (IRR = 0.82, 95% CI 0.79 to 0.84, p < 0.001) during the EVD outbreak 2. Institutional childbirths significantly decreased by 11% (IRR = 0.89, 95% CI 0.87 to 0.91, p < 0.001) during EVD outbreak 3. Postnatal care significantly decreased 22% (IRR = 0.78, 95% CI 0.75 to 0.80; p < 0.001) during the EVD outbreak 4. Maternal mortality ratio at the health facilities significantly increased by 34% (IRR = 1.34, 95% CI 1.07 to 1.69, p = 0.01) during the EVD outbreak, and stillbirth rate significantly increased by 24% (IRR = 1.24, 95% CI 1.14 to 1.35, p < 0.001) during the EVD outbreak	8/9
Leno et al. 2018 [48] Guinea	Cross-sectional study	From January 1, 2013, to December 31, 2014	To compare PMTCT indicators before Ebola (2013) and during Ebola (2014)	1. Pre-EVD the mean ANC1 and above contact was (1617 ± 53) in 2013 versus during EVD 1065 ± 29 in 2014, p = 0.0004 EVD affected areas. ANC contact declined by 41% 2. Pre-EVD the mean ANC1 and above contact was (1817 ± 331) in 2013 versus during EVD (1689 ± 280 in 2014, p = 0.5696) in EVD unaffected areas. ANC1 and above contact declined by 7% (1689 ± 280 in 2014, p = 0.5696) EVD unaffected areas 3. The proportion of HIV+ pregnant women who gave birth at home increased significantly during the EVD (7% in 2013 versus 18% in 2014) EVD affected areas, p < 0.0001 4. Pregnant women tested for HIV significantly decreased (1460 ± 266) in 2013 versus during EVD (717 ± 140 in 2014, p = 0.000) EVD affected areas. Pregnant women tested for HIV did not significantly decrease (1622 ± 247) in 2013 versus during EVD (1379 ± 212, p = 0.1556) in 2014 EVD unaffected areas	7/9
Lori et al. 2015 [39] Liberia	Case series study	January 1–October 30, 2014	To determine women’s maternal health service uptake between January 2012 and October 2014	1. Pre-EVD, average monthly institutional births were 400–500 2. In this study EVD cases increased while the institutional childbirths dramatically decreased by 113 in August 2014	5/9
Ly et al. 2016 [18] Liberia	Cross-sectional study	The pre-EVD period (March 24, 2011–June 14, 2014) and EVD period (June 15, 2014–April 13, 2015)	To estimate the impact of the Ebola outbreak on facility-based birth	1. Pre-EVD outbreak, 686 child births were reported and 212 during the outbreak 2. During EVD outbreak institutional childbirths significantly declined by 30% (AOR = 0.70, 95% CI 0.50–0.98, p = 0.037) Women’s belief that health facilities are or maybe a source of Ebola transmission (AOR = 0.59, 95% CI 0.36 to0.97, p = 0.038)	9/9
Quaglio et al. [53] 2019 Sierra Leone	Prospective observational study	From 2012 to 2018	To determine the trends concerning the utilization of maternal and child health (MCH) services before, during and after the Ebola outbreak	1. Pre-Ebola monthly average ANC1 contact increased by 7 (7, 95% CI 4 to 10, p < 0.001), and ANC4 contact increased by 6 (6, 95% CI 4 to 8, p < 0.001) at community level 2. Pre-Ebola monthly average institutional childbirth increased by 8 (8, 95% CI 6 to 10, p < 0.001) at community level 3. The trends pre-EVD versus during EVD significantly changed ANC1 contact by 74 (74, 95% CI 3 to 145, p = 0.042) and ANC 4 contact by 80 (80, 95% CI 21 to 139, p = 0.008) at community level 4. The trends pre-EVD versus during EVD significantly changed institutional childbirths by 148 (148, 95% CI 99 to 196, p < 0.001) at community level 5. The trends pre-EVD versus post EVD significantly decreased ANC 1 contact by 6 (− 6, 95% CI − 10 to − 3, p < 0.001), ANC 4 by 8 (− 8, 95% CI − 11 to − 5, p < 0.001) at community level 6. The trends pre-EVD versus post EVD institutional childbirth significantly decreased (− 7, 95% CI − 10 to − 4, p < 0.001) at community level 7. Pre-EVD institutional childbirth significantly increased by 11 (11, 95% CI 2 to 21, p = 0.02) at hospital level 8. The trends pre-EVD versus during EVD major obstetric complications significantly changed by 4 (4, 95% CI 1 to 7, p = 0.006) at hospital level 9. The trends pre-EVD versus during EVD institutional childbirth significantly changed by 4 (4, 95% CI 2 to 6, p = 0.001) at hospital level 10. The trends pre-EVD versus during EVD in the reduction of maternal deaths by 1 (− 1,95% CI − 2 to 0, p = 0.042) at hospital level 11. The trends pre-EVD versus post EVD major obstetric complications significantly decreased by 4 (− 4, 95% CI − 7 to − 1, p = 0.009) at hospital level 12.The trends pre-EVD versus post EVD institutional childbirth significantly decreased by 3 (− 3, 95% CI − 5 to − 1, p = 0.001) at hospital level 13. The reduction of maternal deaths pre versus during EVD (-1, 95% CI -2 to 0, p = 0.042) at hospital level	9/9
Shannon et al. 2017 [49] Liberia	Cross-sectional study	Before (July–December 2013), during (July–December 2014) and after (July–December 2015) the EVD outbreak	To determine access to antenatal care (ANC), deliveries and their outcomes before, during and after the 2014–2015 Ebola outbreak	1. ANC1 declined by 14%, and ANC4 and above also declined by 9% in 2014 compared with 2013 2. During EVD, skilled birth attendance declined by 32%, unskilled birth attendance declined by 76%, and caesarean section declined by 60% 3. Pre-Ebola, there were 538 stillbirths from 48,864 total births 4. During Ebola, there were 328 stillbirths from 30,781 total births (RR = 0.61, 95% CI 0.53 to 0.70) 5. Post-Ebola 504 stillbirths from 48,260 total births (RR = 0.60, 95% CI 0.53 to 0.68) 6. Pre-Ebola, there were 276 neonatal deaths from 48,326 live births 7. During Ebola, there were 98 neonatal deaths from 30,453 live births (RR = 0.56, 95% CI 0.45 to 0.71) 8. Post-Ebola, there were 212 neonatal deaths from 47,765 live births (RR = 0.78, 95% CI 0.65 to 0.93) 9. Pre-Ebola, there were 155 maternal deaths from 48,864 live births 10. During Ebola, there were 73 maternal deaths from 30,781 live births (RR = 0.75, 95% CI 0.57 to 0.98) 11. Post-Ebola, there were 130 maternal deaths from 48,260 live births (RR = 0.85, 95% CI 0.67 to 1.07)	6/9
Sochas et al.2017 [51] Sierra Leone	Cross-sectional study	From 2012 to 2015	To quantify the extent of the drop in utilization of essential reproductive, maternal, and neonatal health services	1. Pre-Ebola ANC4 attendance was 74.2%, and during EVD ANC4 attendance decreased by 30.7% 2. During post-EVD ANC4 attendance decreased by 22.2% 3. Pre-Ebola institutional childbirth was 57.4% and during EVD declined by 13.0% 4. During post-EVD institutional childbirth decreased by 8.7% 5. Pre-Ebola PNC was 68.3%, and during EVD, it declined by 19.8% 6. Post-Ebola PNC decreased by 13% 7. Due to the decline of obstetric care, an additional 3593 maternal, neonatal and stillbirth deaths occurred in 2014–2015	8/9
Wagenaar et al. 2018 [50] Liberia	Cross-sectional study	from 1 January 2010 to 31 December 2016	To estimate the immediate and lasting effects of the 2014–2015 Ebola virus disease (EVD) outbreak on public-sector primary healthcare delivery	1.Pre-EVD ANC1 significantly decreased by 30.8% (-30.8,95% CI − 38.4% to − 23.3%, p < 0.001) 2. Early EVD ANC1 contact significantly decreased by 35.2% (− 35.2, 95% CI − 45.8% to − 24.7%, p < 0.001) 3. Post EVD ANC1 contact decreased by 12,426 (-12,426, 95% CI − 53,898 to + 29,546, = 0.558) 4. Pre-EVD institutional births significantly increased by 91.6% (+ 91.6%, 95% CI 95% CI + 61.4% to + 121.9%, p < 0.001) 5.During EVD Institutional births significantly decreased by 5122 (− 5122, 95% CI − 8767 to − 1234, p = 0.003) 6. Post EVD institutional childbirth decreased by 1639 (− 1639, 95% CI − 18,343 to + 16,229, p = .804) 7. Early EVD Postnatal care significantly lessened within 6 weeks of births by 17,191 (− 17,191, 95% CI − 28,344 to − 5,775, p = 0.002) 8. Post EVD PNC significantly declined by 15,144 (− 15,144, 95% CI − 29,453 to − 787, p = 0.040)	9/9

*ANC1* 1st Antenatal care visit (booking visit); *ANC4* four Antenatal care visits; *IRR* incidence rate ratio; *EVD* Ebola Virus Disease; *QS* quality score

#### Operational Definition

Maternal health services are those providing antenatal care, institutional childbirth, and postnatal care.

Antenatal care is the care received by women during pregnancy from skilled health care providers at least once at a health facility.

Institutional childbirth is childbirth attended by skilled health care providers at health facilities.

Postnatal care is received by women from skilled health care providers at health facilities from 48 hours to 6 weeks after childbirth.

Stillbirth is the death of a baby before or during birth after 28 weeks of gestation.

Neonatal death is the death of a baby within the first 28 days of life.

Perinatal mortality is stillbirth plus early (less than seven days) neonatal death.

## Results

Our search strategy retrieved 488 articles from the selected databases that were eligible for first-round screening of titles and abstracts. Thirty-eight articles were excluded due to duplication, 427 articles were excluded based on their titles and abstracts, and the remaining 23 articles were appraised with a full-text screening. Eleven articles were excluded after a full-text review due to unreported maternal health service utilisation. Finally, 12 studies [16–18, 35, 39, 47–53] were included for this systematic review that met the critical appraisal checklists, irrespective of their study design (Fig. 1).

### Characteristics of the included studies

**Fig. 2:**
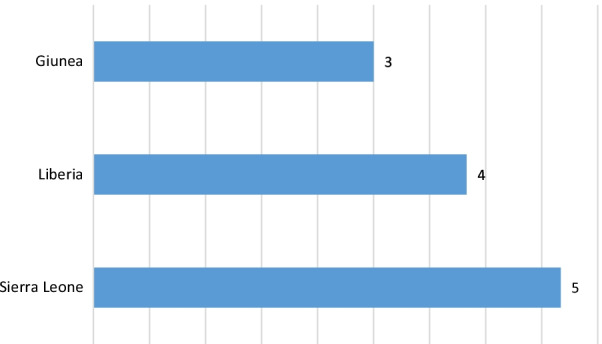
Distribution of selected articles for a systematic review of the effect of Ebola virus disease on maternal and neonatal health services utilisation in West Africa by country

Two-thirds (8 studies) of the included articles were cross-sectional study designs, and the remaining articles were case series, ecological, retrospective cohort and prospective cohort studies. More than 40% (5 studies) of the included articles were published in 2017, one-fourth of the included articles were published in 2016, and all included articles were published within 2015–2019 (Table 2). In addition, the included articles were from 3 West African countries (Guinea, Liberia, and Sierra Leone), 41.7% of the studies were from Sierra Leone [17, 35, 51–53], 33.3% were from Liberia [18, 39, 49, 50] and 25% were from Guinea [16, 47, 48] (Fig. 2).

**Table 2:** Characteristics of included studies for systematic review

Category	Subcategory	Frequency	Percent (%)
Study design	Cross sectional	8	66.7
	Ecological	1	8.3
	Case series	1	8.3
	Retrospective cohort	1	8.3
	Prospective cohort	1	8.3
Year of publication	2015	1	8.3
	2016	3	25
	2017	5	41.7
	2018	2	16.7
	2019	1	8.3

### Antenatal care

Nine studies showed the effect of EVD on antenatal attendance [16, 17, 47–53] (Table 1). ANC attendance in Guinea, Sierra Leone and Liberia significantly decreased during the EVD outbreak. For example, a study conducted at Macenta district Guinea [47] showed that pre-EVD on average 2053 pregnant women attended per month for their first ANC, while pregnant women attendance for their first ANC declined on average per month by 59% (842) and similar trends have happened in ANC 3 and above during EVD outbreak. Post-EVD attending ANC1 increased 1260 per month and recovered by 63%. The recovery gap was (37%, p < 0.001) compared with pre-EVD attending ANC1 (Table1).

In addition, a study conducted at Forest region Guinea [16] revealed that pre-EVD on average attending ANC1 substantially increased per month by 109 (109, 95% CI 54 to 164, p = 0.0005), and attending ANC3 and above also significantly increased by 119 (119, 95% CI 79 to 158, p < 0.0001). However, attending ANC1 on average significantly decreased per month by 418 (− 418, 95% CI − 535 to − 300, p < 0.0001), and attending ANC3 and above also significantly decreased by 363 (− 363, 95% CI − 485 to − 242, p < 0.0001) during the EVD outbreak. The overall trend during versus post-EVD attending ANC1 on average significantly declined per month by 136 (− 136, 95% CI − 231 to − 40; p = 0.0075), but the post-EVD attending ANC3 and above were not significantly different (13, 95% CI − 109 to 134, p = 0.8286) (Table 1).

A similar study conducted in Guinea [48] revealed that, on average, 1617 women attended ANC at least once (ANC1) in 2013 (1617 ± 53), but on average, 1065 women attended ANC1 and above during the EVD outbreak in 2014 (1065 ± 29, p = 0.0004) in EVD affected areas. It indicated that attending ANC1 and above significantly declined in Guinea Ebola-affected areas during the EVD outbreak. On average, 1817 women attended ANC1 and above in 2013 (1817 ± 331), but on average, 1689 women attended ANC1 and above during the EVD outbreak in 2014 (1689 ± 280, p = 0.5696) in EVD unaffected areas. The study also showed that attendance at ANC1 and above did not significantly change in areas that were not affected by Ebola in Guinea (Table 1).

A study conducted in Liberia [50] showed that pre-EVD (January 2010–May 2014), attending ANC1 significantly decreased by 30.8% (95% CI − 38.4%, − 23.3%, p < 0.001). During EVD, attending ANC1 also significantly decreased by 35.2% (95% CI − 45.8% to − 24.7%, p < 0.001). Similarly, attending ANC1 and above significantly decreased by 18% (IRR = 0.82, 95% CI 0.79 to 0.84) in Sierra Leone [17] (Table 1). A study conducted at Pujehun district in Sierra Leone [53] showed that pre-EVD, attending ANC1 were on average seven times more likely to increase per month (7, 95% CI 4 to 10, p < 0.001), and attending ANC4 were six times more likely to increase per month (6, 95% CI 4 to 8, p < 0.001) at the community level. Whereas the trend pre-EVD versus post-EVD attending ANC1 were six times more likely to decrease per month (− 6, 95% CI − 10 to − 3, p < 0.001), and attending ANC4 were eight times more likely to decrease (− 8, 95% CI − 11 to − 5, p < 0.001) at the community level (Table 1).

### Institutional and mode of childbirth

Eleven studies reported the effect of EVD on institutional childbirth [16–18, 35, 39, 47, 49–53] (Table 1). Institutional childbirth in Guinea, Sierra Leone and Liberia significantly decreased during the EVD outbreak compared with the same season pre-EVD. A nationwide study conducted in Sierra Leone [35] revealed that pre-EVD, the number of women attending institutional childbirth was 394. In contrast, institutional childbirth decreased by 28% (283) during EVD.

A study conducted in Sierra Leone [17] showed that institutional childbirth significantly decreased, by 11% (IRR = 0.89, 95% CI 0.87 to 0.91, p < 0.001) during the EVD outbreak. A study conducted at Pujehun district in Sierra Leone [53] showed that pre-EVD, women were on average eight times more likely to attend institutional childbirth per month (8, 95% CI 6 to 10, p < 0.001) at the community level. Post-EVD women were seven times less likely to attend institutional childbirth (− 7, 95% CI − 10 to − 4, p < 0.001) at the community level. Similarly, pre-EVD women were on average eleven times more likely to attend institutional childbirth per month (11, 95% CI 2 to 21, p = 0.02) at the hospital level. Post-EVD, women were four times more likely to attend institutional childbirth (4, 95% CI 2 to 6, p = 0.001) at the hospital level (Table 1).

A study conducted in Forest region Guinea [16] revealed that pre-EVD, institutional childbirth on average had significantly increased by 61 per month (61, 95% CI 38 to 84, p < 0·0001). Institutional childbirth on average significantly decreased by 240 per month (− 240, 95% CI − 293 to − 187; p < 0·0001) during the EVD outbreak. The overall trend in institutional childbirth during EVD versus post EVD was not significantly different (− 30, 95% CI –80, to 20, p = 0·2294) (Table 1).

A study conducted in Rivercess County, Liberia [18] showed that institutional childbirth significantly decreased by 30% during EVD (AOR = 0.70, 95% CI 0.50–0.98, p = 0.037). A nationwide study conducted in Liberia [49] revealed that pre-EVD 6468 births were attended by skilled health care providers, but only 4367 births were attended by skilled health care providers during EVD. These figures demonstrate that institutional childbirth decreased by 32% during EVD (Table 1).

Three studies reported the effect of EVD on caesarean section birth rates [35, 47, 49] (Table 1). Caesarean section childbirth in Guinea, Sierra Leone and Liberia significantly declined during the EVD outbreak. A nationwide study conducted in Sierra Leone [35] showed that pre-EVD caesarean section birth was 112 per week and that caesarean section birth rates decreased by 20% (89) during EVD. In Guinea [47] caesarean section birth rates significantly decreased during EVD. In contrast, full recovery has been noted in the post-Ebola monthly mean of caesarean section (37, SD = 8) compared to the pre-Ebola level (38, SD = 7, p = 0.692). A nationwide study conducted in Liberia [49] revealed that pre-EVD 472 women gave birth by caesarean section while 191 women gave birth by caesarean section during EVD, indicating that caesarean section birth rates declined by 32% (Table 1).

Importantly, five studies reported the effect of EVD on maternal and neonatal mortality [17, 47, 49, 51, 53] (Table 1). Maternal and neonatal mortality rates in Guinea, Sierra Leone, and Liberia decreased during the EVD outbreak. A study conducted in Sierra Leone [17] showed that the maternal mortality ratio at the health facilities significantly increased by 34% (IRR = 1.34, 95% CI 1.07 to 1.69, p = 0.01) during the EVD outbreak, and the stillbirth rate significantly increased by 24% (IRR = 1.24, 95% CI 1.14 to 1.35, p < 0.001) during the EVD outbreak. Notably, a study conducted in Sierra Leone [53] revealed that pre-EVD maternal deaths significantly decreased by 1(− 1, 95% CI − 2 to 0, p = 0.042) at the hospital level compared with EVD outbreak. Another study conducted in Sierra Leone [51] showed that an additional 3593 maternal deaths, neonatal deaths, and stillbirth occurred in 2014–2015 (Table 1).

Furthermore, a study conducted in Guinea [47] showed maternal deaths were low and remained similar across pre, during and post EVD (0.1–0.2%, p > 0.05). Similarly, stillbirths were low and remained similar across pre, during and post-EVD. Neonatal deaths were also low pre and during (range 1.1–1.7%) but were higher in the post-Ebola period compared to the pre-Ebola period (p < 0.01).

A study conducted in Liberia [49] revealed that maternal deaths decreased by 25% during EVD ((RR = 0.75, 95% CI 0.57 to 0.98). Whereas post EVD maternal deaths increased by 15%  (RR = 0.85, 95% CI 0.67 to 1.07) and similar with pre EVD. Stillbirth increased by 39% during EVD (RR = 0.61, 95% CI 0.53 to 0.70). Similarly, stillbirth increased by 40% post EVD (RR = 0.60, 95% CI 0.53 to 0.68). Neonatal deaths decreased by 44% during EVD (RR = 0.56, 95% CI 0.45 to 0.71), but neonatal deaths decreased by 22% post EVD (RR = 0.78, 95% CI 0.65 to 0.93) compared with pre-EVD. This number showed that neonatal deaths were lower during EVD than pre EVD and post EVD (Table 1).

### Postnatal care

Four studies reported the effect of EVD on institutional childbirth [17, 50–52] (Table 1). PNC in Sierra Leone and Liberia decreased during the EVD outbreak. A study conducted in Liberia [50] showed that during EVD, postnatal care significantly decreased within six weeks of births by 17,191 (− 17,191,95% CI − 28 344 to − 5775, p = 0.002) and noticeably PNC significantly decreased post-EVD by 15,144 (− 15,144, 95% CI − 29,453, − 787, p = 0.040). A study conducted in Sierra Leone [17] also revealed that attending PNC decreased by 22% during EVD (IRR = 0.78, 95% CI 0.75 to 0.80). In Sierra Leone [51], a study showed that pre-EVD PNC utilisation was 68.3%. Whereas PNC decreased by 19.8% during the outbreak and recovered 13% post-EVD (Table 1).

## Discussion

The purpose of this systematic review was to identify, appraise and synthesise studies that reported the effect of EVD on maternal health service utilisation in West Africa. This systematic review showed that antenatal care, institutional childbirth, and postnatal care significantly decreased during EVD in three countries (Guinea, Liberia, and Sierra Leone). This review included three articles from Guinea, four articles from Liberia, and five articles from Sierra Leone. Institutional childbirth was reported in eleven studies, caesarean section and postnatal care were each reported in three studies. Within countries, institutional childbirth was reported in five studies in Sierra Leone, ANC was reported in four studies in Sierra Leone, PNC was reported in three studies in Sierra Leone, and PNC wasn't reported in Guinea. Importantly, this systematic review presented maternal health services utilisation and perinatal outcomes pre-EVD, during EVD and post-EVD.

Among the findings, studies conducted in Guinea [16] and Sierra Leone [53] showed that pre-EVD, women were more likely to attend ANC1 and above. This finding is consistent with studies performed in Liberia [54], Sierra Leone [55], and Guinea [56]. Obstetric care providers, governments, maternal and child health advocates of these countries implemented effective interventions pre-EVD, for example, providing free health services [22] to improve ANC utilisation to reduce maternal and neonatal mortality [57].

The current review showed that in Guinea [16, 47] and Liberia [49, 50] showed significantly decreased in attending ANC1 and above during EVD, while one study in Sierra Leone [52] showed that attending ANC1 and above had no significant difference pre and during Ebola outbreak. This study was conducted in a rural district that experienced low Ebola cases than other areas. This finding is consistent with a study conducted in Taiwan [58] on SARS-1 and a review in West Africa [31], and a systematic review on Ebola [59]. The reduction of attending ANC 1 and above could be due to the absence of health care providers, a shortage of personal protective equipment, women beliefs that hospitals are exposure centres, the shutdown of some health institutions, the health care providers contracting Ebola virus and death [60], and/or distrust between health care providers and the community [61].

Notwithstanding, there was no significant change during EVD in the unaffected areas in Guinea [48]. Studies in Guinea [16, 47] and Sierra Leone [53] showed that ANC utilisation also significantly increased post-EVD but did not reach pre-EVD level. These findings suggest that EVD disturbed ANC services. To curb these problems, the international community, responsible bodies, and health care providers need to implement extraordinary interventions tailored to the local community to achieve Sustainable Development Goal 3 by 2030.

Institutional childbirth before EVD significantly increased in Guinea [16], Liberia [50], and Sierra Leone [53], while other studies in Guinea [47] and Liberia [18, 39] showed that institutional childbirth increased to some extent before EVD. These findings are similar to those of studies performed in Liberia [54], Sierra Leone [55], and Guinea [56]. In addition, studies conducted in Guinea [16], Liberia [18, 50], and Sierra Leone [17, 53] showed that women were less likely to have an institutional birth during EVD. These findings coincide with a study conducted in Taiwan [58], a study conducted in West Africa [14, 31] and a systematic review of the effect of Ebola on pregnancy and breast-feeding mothers [59]. These interruptions could be lack of transport due to lockdown, loss of income, lower health-seeking behaviour due to EVD, community mistrusted the health care providers and the health system [62] and shut down some health facilities [35].

Importantly, post-EVD women were more likely to have an institutional birth, indicating a recovery in Guinea [16, 47], Liberia [50] and Sierra Leone [53]. One study found the rate had returned to the pre-EVD period [16], while the others [47, 50, 53] had not fully recovered to the pre-EVD level of utilisation of institutional childbirth. The institutional childbirths could have recovered due to the resilience of the maternal health system, which is based on trust built between the community and health care providers, the capability of the healthcare facilities and deploying additional obstetric care providers in the highly EVD affected areas.

Caesarean section birth rates in Guinea [47], Sierra Leone [35] and Liberia [49] significantly declined during the EVD outbreak. In a nationwide study conducted in Sierra Leone, caesarean section decreased by 20% during EVD. Similarly, a nationwide study in Liberia showed that caesarean section decreased by 32% during EVD. Besides, a study conducted in Guinea showed that caesarean section births significantly decreased during EVD. In contrast, full recovery has been noted in the post-Ebola monthly mean of caesarean section (37, SD = 8) compared to the pre-Ebola level (38, SD = 7, p = 0.692). This finding is consistent with a study conducted in West Africa [14].

The current systematic review also showed that during EVD, there were higher rates of maternal mortality in Sierra Leone [17, 51], but during EVD, maternal mortality rates were lower than before EVD in Guinea [47] and Liberia [49]. Similarly, stillbirth and neonatal deaths were less likely to occur during EVD [49]. This decrease might be due to the shutdown of some health facilities, under-reporting or reporting errors, and increasing numbers of home birth. Moreover, this systematic review showed that during EVD, PNC also significantly decreased in studies performed in Liberia [50] and Sierra Leone [17]. Decrease in PNC might be due to fear of acquiring EVD by postpartum mothers, their family and health care providers’ and a resulting pressure to discharge early. Contributing factors included economic recession, lack of transportation [62] and shutdown of health facilities [35] due to EVD.

This review has synthesised the current evidence of the effect of EVD on maternal health service utilisation and perinatal outcomes during EVD and post-EVD. Strengths of the study include that it systematically synthesised evidence on the effect of EVD on ANC, institutional childbirth, PNC, and perinatal outcomes. Data extraction and evidence synthesis were done by three reviewers, to strengthen the reliability of the study outcomes and minimise the subjectivity of evidence synthesis and interpretation. The quality of included studies was appraised using the Joanna Brigg's Institute (JBI) validated quality appraisal method [44]. All studies included in this paper indexed in Medline, PubMed, CINAHL, Scopus, EMBASE and African journals online databases and published in English. Besides these strengths, the limitation of the systematic review a study focused on the access of ANC, institutional childbirth, PNC, and perinatal outcomes rather than on the quality of ANC, institutional childbirth, and PNC. Quality of ANC, institutional childbirth, and PNC are useful to consider, as access to poor quality services could increase maternal and perinatal morbidity and mortality. Data collection carried out after the outbreak ended is subject to social desirability and recall bias. Other limitations are lack of coverage of ANC, institutional childbirth, and PNC due to having no population-level denominators. Most of the data has been taken from records; there are under or overestimated data.

## Conclusion

Based on our findings, rates of antenatal care, institutional childbirth, and postnatal care were attempting to recover post-Ebola virus disease. We have three recommendations based on the synthesis of this review. Firstly, responsible bodies and stakeholders need to prepare locally tailored interventions to increase the number of women attending ANC, institutional childbirth, and PNC services post-EVD and future outbreaks including COVID-19. Secondly, in order to build trust, creating community networks between health care providers and trusted community leaders may increase the number of women attending ANC, institutional childbirth, and PNC services post-EVD and during future outbreaks. Thirdly, governments and stakeholders need to establish a non-epidemic task force that provides equipment and monitors maternal health services to sustain services post- EVD and during future outbreaks. Further rigorous studies are needed to examine health centre and hospital availability, accessibility, and capacity to deliver maternal health services.

## Data Availability

Most of the data analysed during the systematic review are included in this manuscript.

## Abbreviations

ANC: Antenatal care
EVD: Ebola virus disease
PNC: Postnatal care
WHO: World Health Organization
